# Echographic and Kinetic Changes in the Shoulder Joint after Manual Wheelchair Propulsion Under Two Different Workload Settings

**DOI:** 10.3389/fbioe.2014.00077

**Published:** 2014-12-23

**Authors:** Ángel Gil-Agudo, Marta Solís-Mozos, Beatriz Crespo-Ruiz, Antonio J. del-Ama Eng, Enrique Pérez-Rizo, Antonio Segura-Fragoso, Fernando Jiménez-Díaz

**Affiliations:** ^1^Biomechanics and Technical Aids Unit, Department of Physical Medicine and Rehabilitation, National Hospital for Paraplegics, Servicio de Salud de Castilla-La Mancha, Toledo, Spain; ^2^Health Sciences Institute of Castilla-La Mancha, Toledo, Spain; ^3^Laboratory of Performance and Sports Rehabilitation, Faculty of Sport Science, University of Castilla-La Mancha, Toledo, Spain

**Keywords:** kinetics, shoulder injury, wheelchair propulsion, biomechanics, ultrasonography, spinal cord injury

## Abstract

Manual wheelchair users with spinal cord injury (SCI) have a high prevalence of shoulder pain due to the use of the upper extremity for independent mobility, transfers, and other activities of daily living. Indeed, shoulder pain dramatically affects quality of life of these individuals. There is limited evidence obtained through radiographic techniques of a relationship between the forces acting on the shoulder during different propulsion conditions and shoulder pathologies. Today, ultrasound is widely accepted as a precise tool in diagnosis, displaying particularly effectiveness in screening the shoulder rotator cuff. Thus, we set out to perform an ultrasound-based study of the acute changes to the shoulder soft tissues after propelling a manual wheelchair in two workload settings. Shoulder joint kinetics was recorded from 14 manual wheelchair users with SCI while they performed high- and low-intensity wheelchair propulsion tests (constant and incremental). Shoulder joint forces and moments were obtained from inverse dynamic methods, and ultrasound screening of the shoulder was performed before and immediately after the test. Kinetic changes were more relevant after the most intensive task, showing the significance of high-intensity activity, yet no differences were found in ultrasound-related parameters before and after each propulsion task. It therefore appears that further studies will be needed to collect clinical data and correlate data regarding shoulder pain with both ultrasound images and data from shoulder kinetics.

## Introduction

Manual wheelchair users with spinal cord injury (SCI) have a high prevalence of shoulder pain (Bayley et al., 1987; Sie et al., 1992; Subbarao et al., 1995; Escobedo et al., 1997; Curtis et al., 1999; Ballinger et al., 2000; Boninger et al., 2001; Mercer et al., 2006), with estimates ranging from 30% (Ballinger et al., 2000) to 73% (Pentland and Twomey, 1991). As the life expectancy of patients with SCI continues to increase, the prevalence of shoulder impingement related to damage of the rotator cuff is rising (Bayley et al., 1987). Since wheelchair users depend strongly on the upper extremity for independent mobility and their daily activities, shoulder pain has a strong negative impact on their quality of life.

The shoulder joint experiences a repetitive and continuous load during the push phase of the wheelchair propulsion cycle. Since the upper limb is not specialized for this action, this repetitive loading may cause musculoskeletal disorders at the shoulder joint, predisposing manual wheelchair users to upper limb pathologies (Bayley et al., 1987). Indeed, this mechanical stress leads to overuse syndrome, which is a possible factor influencing the development of shoulder pain in this population and commonly, injuries of the rotator cuff (Subbarao et al., 1995). High-intensity wheelchair propulsion increases upward shoulder joint forces, which could result in upward translation of the humeral head and subsequent compression of the subacromial structures against the overlying acromion (Kulig et al., 1998). Repetitive strains of rotator cuff tendons can potentially induce microinjuries, which may facilitate tendon degeneration. Therefore, it is important to define the biomechanical factors that may predispose wheelchair users to shoulder pathologies in order to recommend interventions that minimize the shoulder load during propulsion (Rodgers et al., 1994; Kulig et al., 1998; Cooper et al., 1999; Finley et al., 2004; Mulroy et al., 2005; Mercer et al., 2006; Moon et al., 2013), contemplating different lesion levels (Gil-Agudo et al., 2010a). Recommendations to prevent shoulder injury based solely on pushrim biomechanics are available (Boninger et al., 2005). However, research using inverse dynamics techniques revealed that posterior and superior forces both act on the shoulder joint during the push phase of propulsion, these probably being related to coracoacromial ligament edema and compression of the rotator cuff, respectively (Koontz et al., 2002; Van Drongelen et al., 2005; Mercer et al., 2006; Collinger et al., 2008; Gil-Agudo et al., 2010a).

Despite the logic of these claims, there is limited radiographic evidence for the relationship between shoulder joint forces in different propulsion conditions and shoulder pathologies. Several radiographic abnormalities have been reported in the shoulder of the SCI population (Bayley et al., 1987; Wylie and Chakera, 1988; Boninger et al., 2001; Kivimäki and Ahoniemi, 2008; Akbar et al., 2010). Acute changes in the shoulder tendons upon high-intensity wheelchair propulsion may contribute to the pathological process that leads to a chronic pathology and pain (Van Drongelen et al., 2007). Indeed, acute exercise induces changes in tendon metabolism and increased inflammation (Landberg et al., 1999). Nevertheless, to the best of our knowledge only one previous study has addressed the relationship between kinetics and shoulder pathologies (Mercer et al., 2006), assessing the shoulder pathology by magnetic resonance imaging (MRI). However, MRI is a complex technique that is not easy to conduct immediately after propelling the wheelchair. In fact, musculoskeletal ultrasound techniques have several advantages over MRI when diagnosing shoulder pathologies, such as portability, ease to implement in clinics, and the capability to assess joint dynamics during motion. Thus, this technique allows the shoulder joint to be readily assessed immediately before and after conducting a propulsion test in laboratory settings. Moreover, ultrasound is a technique that is widely available in clinical settings due to its diagnostic precision (Landberg et al., 1999; Teefey et al., 2004; Iannotti et al., 2005) and it is particularly effective in assessing the shoulder rotator cuff (Allen, 2008). The acute changes in shoulder tendons that might follow strong demands on propulsion could contribute to chronic shoulder pathologies and pain. Such acute changes can be rapidly screened using ultrasound immediately after completing the propulsion task in a controlled environment.

Tangential forces acting on the hand rim have been shown to be directly linked to net shoulder moments, indicative of a higher risk of shoulder injury (Koontz et al., 2002; Desroches et al., 2008). Therefore, it can be assumed that the greater the demand on propulsion, the higher the net shoulder moments, and hence, the risk of shoulder injury increases. Acute changes in shoulder tendons have been studied previously by ultrasound after two different high-intensity propulsion activities. In both cases, ultrasound findings were not correlated with kinetic data from the shoulder joint, probably because these measurements were not made in the two different intensity and standardized exercises employed (Van Drongelen et al., 2007; Collinger et al., 2010).

We hypothesize here that (1) shoulder joint forces would be greater in the more intensive propulsion task and cuff rotator tendon ultrasound changes would be consequently more notable; and (2) it would be possible to establish a link between shoulder joint kinetics and ultrasound-derived metrics. Accordingly, the aim of this study was to compare shoulder joint forces and moments between early and late propulsion instances in two different propulsion protocols: low- and high-intensity activities. In addition, we set out to compare changes in the shoulder evident by ultrasound after performance of the same two different wheelchair propulsion protocols.

## Materials and Methods

### Subjects

Subjects were recruited from the discharge records from a monographic in-patient SCI hospital, sending a letter inviting them to participate in the research study. For inclusion in the study, subjects had to have traumatic SCI at level T2 or below, with AIS grade A or B (Marino et al., 2003), which occurred after the age of 18 and before 45, and with an evolution longer than 18 months at time of the study. Volunteers must use manual wheelchairs as their primary means of mobility. Subjects were excluded if they had had fractures or dislocations in the non-dominant shoulder at any time, upper limb pain that prevented them from propelling a manual wheelchair, progressive or degenerative disability, or a history of cardiopulmonary disease. This study was approved by the ethics review board and all the participants signed an informed consent form prior to enrollment.

### Instrumentation

**Figure 1:**
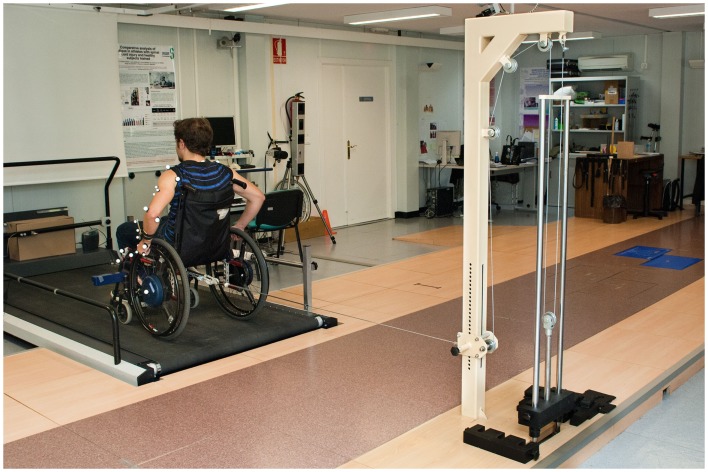
**Overview of the test set-up where the subject is working against extra resistance applied through a pulley system and including the positions of the markers**.

A standard adjustable wheelchair (Action3 Invacare, Invacare Corp, Elyria, OH, USA), was properly fitted for each subject and placed on a treadmill (Bonte Zwolle B.V., BO Systems, Netherlands). A force transducer (Revere ALC 0.5, Vishay Revere Transducers BV, Breda, The Netherlands) was situated in front of the treadmill in order to estimate the rolling resistance, and a custom dead weight and pulley system that can be attached to the back of the wheelchair (van der woude et al., 1986; Van Drongelen et al., 2013) (Figure 1) was also available to regulate the propulsion power output (see below). Propulsion trials were conducted using a safety system, which prevented lateral movements.

Non-dominant upper limb kinematic data were collected at 50 Hz (maximum recording frequency) using passive markers and four camcorders (Kinescan-IBV, Instituto de Biomecánica de Valencia, Valencia, Spain). All subjects were right-hand dominant so that the left upper limb was analyzed and spatial marker coordinates were smoothed out using a procedure of mobile means. Reflective markers were positioned following ISB recommendations to define local reference systems on the hand, forearm, and arm (Wu et al., 2005). The local trunk reference system was defined using markers placed on the seventh cervical vertebra (C7), and on the right (ACRR) and left (ACRL) acromioclavicular joints [the axes of this reference system have been described previously (Gil-Agudo et al., 2010b)]. Markers were also placed on the wheel hub during data collection.

Both wheels of the chair were replaced by two SMART^Wheels^ (Three Rivers Holdings, LLC, Mesa, AZ, USA) to balance the inertial characteristics of both axes and ensure symmetrical propulsion. A synchronization pulse from the Kinescan-IBV was used to trigger the start of the kinetic and kinematic data collection. Kinetic data were recorded at a frequency of 240 Hz and filtered using a Butterworth, fourth-order, low-pass filter with a cutoff frequency of 20 Hz and a zero phase lag. Spatial marker coordinates were interpolated by cubic spline to synchronize with the kinetic data.

### Data collection

Upon arrival at the laboratory, the participants provided their demographic information and a physical examination was performed that included a study of the range of shoulder movement and that identification of the painful point. A visual analog scale (VAS) was used to measure current pain, with 0 indicating a painless shoulder and 100 indicating an intensely painful shoulder. Functional status was assessed using the wheelchair user’s shoulder pain index (WUSPI) (Curtis et al., 1995). Subjects then underwent a base-line ultrasound screening of the non-dominant shoulder before completing the wheelchair propulsion test and ultrasound screening immediately after finishing it.

All subjects performed two different wheelchair propulsion tests, one at high intensity with an incremental workload (Protocol A) and another at low intensity with a constant workload (Protocol B). In order to comply with recommendations on resting periods described in physiology studies (Schuenke et al., 2002), the tests were performed with at least 48 h difference, thereby ensuring complete recovery of the patient. Movements like turning or going backwards were excluded because these could not be performed in the same experimental set up. The four camcorders were fixed above the treadmill, and hence, manual wheelchair propulsion was the only movement that could be registered in such conditions.

To simulate the conditions of wind resistance, the treadmill slope was fixed at 0.7° for both protocols (Mason et al., 2014). The order in which the tests were performed was randomized for each subject, and before testing the subjects were allowed to familiarize themselves with the wheelchair and the experimental set up. Afterwards, the individual rolling resistance was determined in a separate drag test (Marino et al., 2003; Mason et al., 2014). The mean and SD of the friction coefficient was 0.010 ± 0.002, which falls among optimal limits set by previous studies (van der woude et al., 1986; de Groot et al., 2006) regardless of the constraints in lateral movements imposed by our safety system.

Once the rolling resistance was determined, the propulsion power output could be regulated by an additional external force that acted via a pulley system on the wheelchair-user combination (Figure 1). The propulsion power output (PO external) was calculated as Power (W) = Force (N) × Speed (km h^−1^), and the minimum load imposed by the pulley system was 20 W. The speed necessary to adjust the resistance power of each subject to 20 W was therefore calculated using the sum of *F*_drag_ and the dead weight acting via the pulley system (*F*_additional_). Therefore, by varying the dead weight acting through the pulleys and/or the speed of the treadmill, the PO external could be set to a desired value, independently of the experimental subject.

The treadmill speed in protocol A was calculated in order to set the PO external for all subjects at 20 W. Discrete increases of 5 W were introduced every 2 min without rest between stages using the dead weights in the pulley system. The trial was finished either when the subject was exhausted and could not propel the wheelchair any longer or when the security system stopped the propulsion. The maximum criteria were then obtained following the ACSM guidelines (ACSM, 2006).

In protocol B, the treadmill speed was also adjusted to a PO external of 20 W for all subjects, and it remained constant during this protocol. The maximum test duration was fixed at 20 min and the test terminated when the subject stopped propelling the wheelchair or the time limit was reached. A subjective perception of fatigue (Borg scale) was recorded immediately after completing each protocol (Borg, 1970).

### Measures of shoulder pathology

The same physician conducted a physical examination on all the subjects that focused on shoulder injury, as reported previously (Boninger et al., 2001). All ultrasound screenings were also performed by the same physician, who has more than 15 years of training and experience in musculoskeletal ultrasound. Ultrasound was performed with a General Electric Healthcare (Logiq S8) apparatus and using 8–12 MHz linear array transducer. Images of the long head of the biceps tendon and supraspinatus tendon were captured before and immediately after the propulsion task. During the base-line ultrasound examination, external reference landmarks were taped to the shoulder skin. These were not removed until the end of the second propulsion task, allowing ultrasound measurements at the two different time points to be obtained with minimal variation in transducer location, making the procedure more reliable (Collinger et al., 2009). The protocol used in both ultrasound examinations to examine the structures in the shoulder was the same and it was based on previously described techniques (Mack et al., 1985; Middleton et al., 1986; Crass et al., 1987; Middleton, 1992). To examine the transverse image of the biceps tendon, the subject’s hand was placed on their thigh with the palm facing upwards. Supination of the hand with external rotation of the shoulder improved the visualization of the bicipital groove. The transducer was then turned 90° to obtain the long-axis image of the biceps tendon. The supraspinatus tendon was observed with the hand placed behind the back with the shoulder in internal rotation. The acromio-humeral distance was recorded with the arm in internal rotation.

### Data analysis

#### Biomechanical data

The total pushrim force (Ftot) was calculated as the vector sum of the SMART^Wheel^ components (Fx, Fy, Fz). Mechanical effective force (MEF) was calculated as the proportion of the force at the pushrim that contributes to the forward motion (Ft^2^/Ftot^2^), where Ft is the tangential force obtained by dividing the measured mean propulsion moment around the wheel axle by the radius of the pushrim. These kinetics parameters were only calculated over the push phase of the stroke (Koontz et al., 2005).

We used an inverse dynamic model described previously to calculate the shoulder joint forces and moments (Gil-Agudo et al., 2010b). The model was used to calculate the net shoulder joint forces and moments from segment kinematics, the forces acting on the pushrim, and the subject’s anthropometric measurements (Clauser et al., 1969). Net joint forces and moments were calculated on a global reference system and then expressed through the joint reference system (Cooper et al., 1999; Mercer et al., 2006). The analysis focused on the glenohumeral joint, and movements of the scapula, clavicle, and thoracic spine were not considered. The forces reported constituted the reaction forces on the joint and moments were reported as the action moments.

In order to obtain the biomechanical data as close as possible to ultrasound examination, the first and last 20 s of each test were recorded for analysis. For protocol A, the last 20 s corresponded to the maximum step achieved. For protocol B, it corresponded to the last 20 s before finishing the test.

Five consecutive cycles were selected from the 20 s recording of data, and the cycles were normalized from 0 to 100% since the time spent in each cycle varied between individuals and cycles. The push phase started/finished at the instant at which the propulsive moment exerted by the user during hand contact with the pushrim was higher/lower than 1 Nm. The peaks were determined for each stroke individually and then averaged over five cycles. The output variables of the biomechanical model were the time-varying 3D joint net forces and moments. The following sign convention was used:

Forces
  - Fx: +anterior, −posterior.
  - Fy: +superior, −inferior.
  - Fz: +lateral, −medial.

Moments
  - Mx: +adduction, −abduction.
  - My: +internal rotation, −external rotation.
  - Mz: +flexion, −extension.

#### Ultrasound data

**Figure 2:**
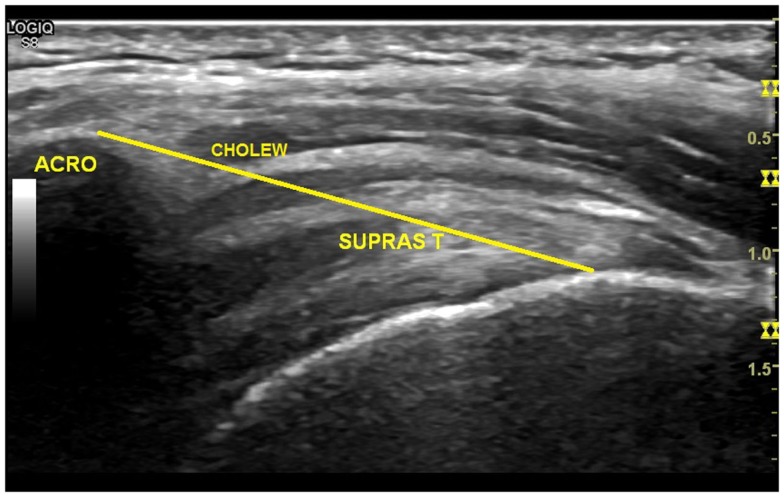
**Measurement of the greater acromion tuberosity distance (Cholewinski index)**.

**Figure 3:**
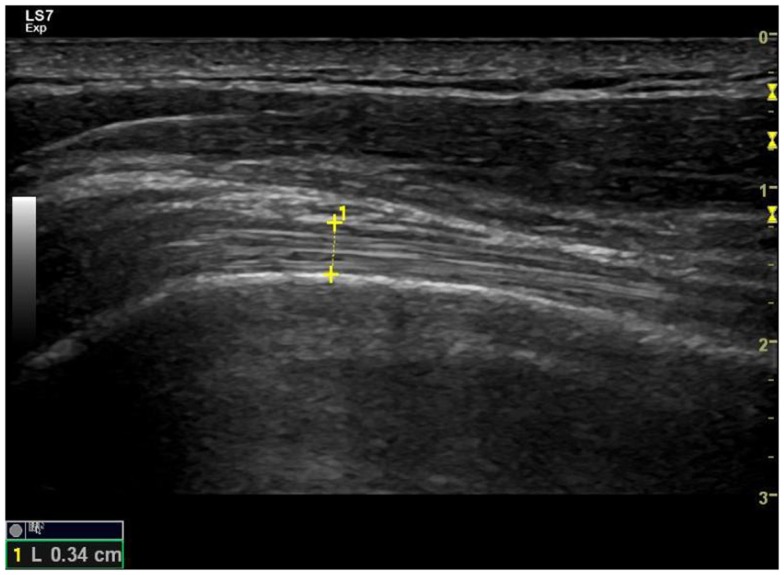
**Long-axis ultrasound examination which shows the fibrillar pattern of the long-biceps tendon**.

**Figure 4:**
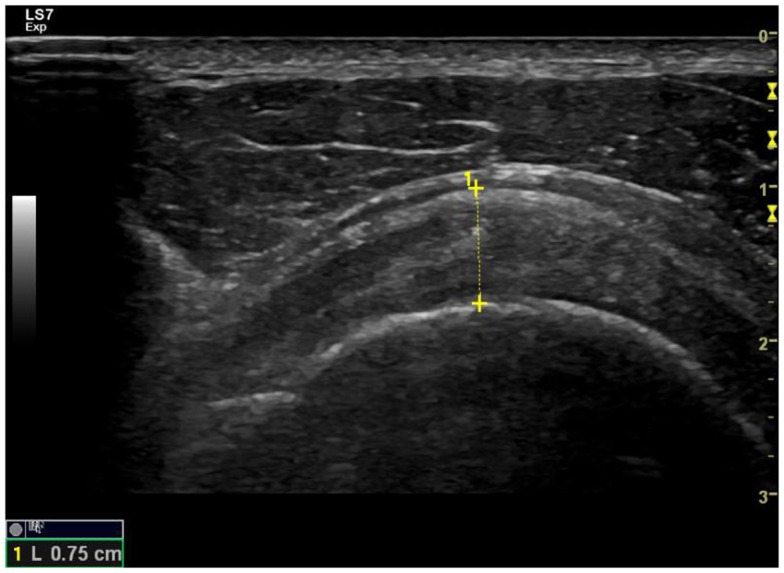
**Examination of the short axis of the supraspinatus tendon to measure its thickness and check the hyperechoic fibrillar pattern**.

The ultrasound images were screened by two reviewers to assess their usability. The anatomical shoulder references, and the biceps and supraspinatus tendon characteristics, were analyzed with custom software written in Matlab (The Mathworks Inc., Natick, MA, USA). The most common ultrasound finding related to the shoulder of SCI manual wheelchair users is an increase in the glenohumeral joint space (Kivimäki and Ahoniemi, 2008), and the most common ultrasound finding after high-intensity wheelchair propulsion activity is an increase in the biceps tendon diameter (Van Drongelen et al., 2007). Nevertheless, a comprehensive analysis of shoulder ultrasound parameters was carried out, including anatomical shoulder references such as acromioclavicular distance (ACD) and acromio-humeral distance using the Cholewinski (CHI) method (acromion to greater tuberosity of humerus) (Seitz and Michener, 2010) (see Figure 2). Several tendon characteristics, such as long-axis biceps tendon thickness (LBTT), long-axis biceps sonoelasticity (LBS), short-axis supraspinatus thickness (SST), and short-axis supraspinatus sonoelasticity (SSS) were also analyzed (Farin et al., 1995; Turrin and Capello, 1997; Park and Kwon, 2011) (Figures 3 and 4). In the longitudinal images of the biceps tendon, a 2 cm length was selected by the researcher that included the part of the tendon located inside the bicipital groove, and the average diameter of this selection was calculated (Van Drongelen et al., 2007).

#### Statistics

Descriptive analysis, including the means and SD for the continuous variables, was performed initially to describe the subject’s characteristics. Differences in the shoulder joint forces, moments, and ultrasound parameters between the two wheelchair propulsion tests were analyzed and all statistical analysis was carried out using SPSS^®^ V.17 for Windows (SPSS Inc., Chicago, IL, USA).

Peak shoulder forces and moments were averaged to create a representative value for each direction. Shoulder joint kinetics was calculated as the average of the peak force or moment for the two wheelchair propulsion test. Differences between early and late propulsion for each protocol and between protocols were analyzed. In order to calculate the differences in shoulder joint forces and moments between both conditions, a Shapiro–Wilk test was applied to the normal distribution of the sample. A Student’s *t*-test for independent samples was applied to those variables that followed a normal distribution. A Mann–Whitney *U* test for independent samples was used to compare those variables that showed a non-parametric distribution. Additionally, correlations between ultrasound parameters and shoulder kinetic data were evaluated using Spearman’s rho. These correlations were performed considering differences obtained in ultrasound and kinetic examinations before and after each protocol. Significance level was set at *p* < 0.05.

## Results

### Subjects

Fourteen subjects with SCI participated in this study, all males. They had an average height of 1.77 m (SD = 0.07; range 1.67–1.87) and weight 68.3 kg (SD = 8.96; range 53–87), and their average age was 35.2 years (SD = 6.11; range 25–43) with an average time since injury of 90.2 months (SD = 54.78; range 37–282: Table 1). As only half of subjects suffered from shoulder pain, we considered all SCI subjects as a single group rather that conducting a separate analysis for those who referred to shoulder pain.

**Table 1:** **Subject’s characteristics, mean (SD)**.

Characteristics	SCI subjects		
*n*	14		
Sex (male/female)	14 male		
Age (years)	35.2 (6.11)		
Weight (kg)	68.3 (8.96)		
Height (m)	1.77 (0.07)		
Time since injury (months)	90.2 (54.78)		
Shoulder pain (no pain/pain)	7/7		
WUSPI (0–150)	25.46 (25.75)		
	Subjects with non-pain: 5.7 (4.98)		
	Subjects with pain: 45.23 (22.37)		
VAS (0–100)	53.8 (5.03)		
	Pain: 74.3 (5.21)		
	Non-pain: 21.4 (4.32)		
Level of injury	D2–D6	D7–D11	D12–L3
	7	2	5

### Biomechanics

The performance of the subjects in both the protocols was considered and the effective mechanical force was similar in both protocols (Table 2), although the increase in the forces and moments was greater after protocol A (high intensity). Considering only protocol A, significant differences were found between early and late propulsion for all the parameters analyzed, except for the adduction and abduction shoulder peak moments (Table 3).

**Table 2:** **Performance in both protocols, mean (SD)**.

	Test duration (min)	Speed (km/h)	Power output (W)	Increasing steps (kg)	Borg scale (0–20)	Mechanical effective force (*N*)
High-intensity task
SCI subjects	14.85 (2.17)	1.44 (0.08)	53.21 (4.20)	1.24 (0.10)	17.42 (1.01)	0.84 (0.11)
Low-intensity task
SCI subjects	20	1.47 (0.08)	20		8.46 (1.94)	0.85 (0.08)

**Table 3:** **Raw mean of the biomechanical variables in the two wheelchair propulsion tasks, mean (SD)**.

		High-intensity task			Low-intensity task		
		Early propulsion	Late propulsion	*p*-Value	Early propulsion	Late propulsion	*p*-Value
Fx (N) (+anterior, −posterior)	Max	41.89 (9.32)	51.28 (10.13)	**<0.05**	43.42 (9.83)	41.38 (10.30)	0.59
	Min	−44.00 (8.04)	−82.14 (18.49)	**<0.01**	−42.54 (9.22)	−45.24 (10.43)	0.47
Fy (N) (+superior, −inferior)	Max	−0.45 (9.33)	21.07 (21.91)	**<0.01**	0.47 (9.91)	−0.12 (11.81)	0.88
	Min	−47.45 (11.60)	−67.16 (21.96)	**<0.01**	−45.35 (8.66)	−49.44 (10.05)	0.25
Fz (N) (+lateral, −medial)	Max	13.84 (5.27)	19.42 (8.39)	**<0.05**	16.29 (7.37)	17.51 (9.57)	0.70
	Min	−9.93 (3.53)	−15.36 (6.72)	**<0.05**	−11.71 (5.25)	−10.98 (2.91)	0.65
Mx (N⋅m) (+adduction, −abduction)	Max	3.08 (1.53)	6.10 (5.83)	0.07	3.35 (2.47)	3.03 (1.90)	0.71
	Min	−5.43 (1.93)	−7.71 (4.15)	0.07	− 4.94 (1.16)	− 5.14 (1.68)	0.71
My (N⋅m) (+int. rotation, −ext. rotation)	Max	2.45 (0.93)	4.65 (1.99)	**<0.01**	2.60 (1.31)	2.58 (1.25)	0.96
	Min	−3.09 (1.29)	−5.23 (2.71)	**<0.05**	−3.19 (0.99)	−3.22 (0.83)	0.93
Mz (N⋅m) (+flexion, −extension)	Max	13.16 (2.79)	24.84 (7.25)	**<0.01**	13.26 (3.10)	14.08 (3.45)	0.51
	Min	−7.09 (2.39)	−11.70 (7.18)	**<0.05**	−7.34 (2.09)	−7.96 (2.30)	0.45

*Bold font indicates statistical significance at *p* < 0.05*.

**Figure 5:**
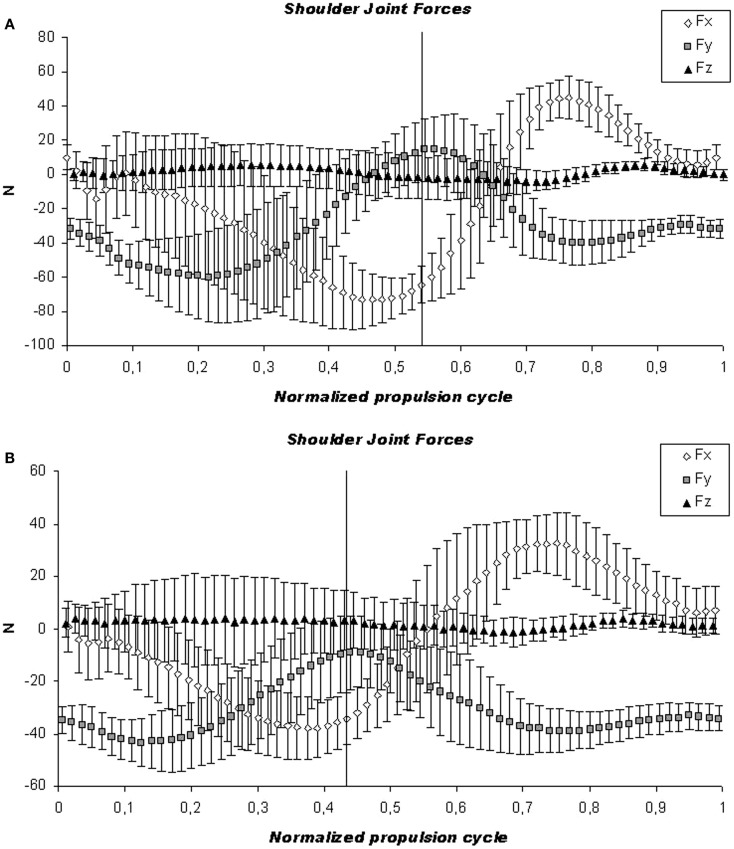
**Kinetic analysis**. Illustration of the mean cycle of shoulder joint forces for the group analyzed. The mean (continuous line) and SD (dashed line) are shown for the high-intensity **(A)** and low-intensity protocol **(B)**. The vertical line indicates the push and recovery phase of the forces on the shoulder joint.

**Figure 6:**
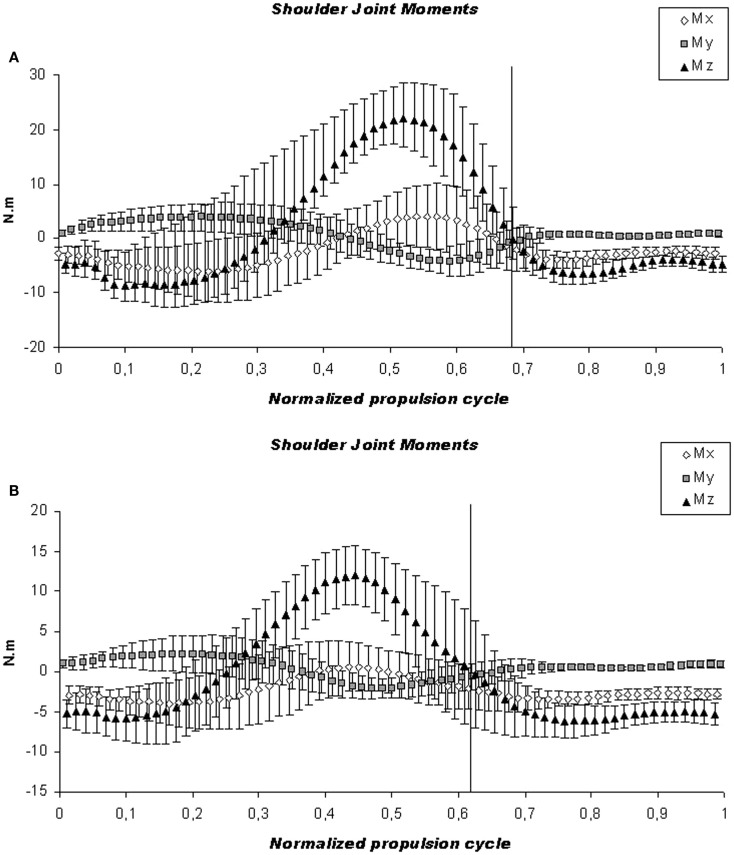
**Kinetic analysis**. Illustration of the mean cycle of shoulder joint moments for the group analyzed. The mean (continuous line) and SD (dashed line) are shown for the high-intensity **(A)** and low-intensity protocol **(B)**. The vertical line indicates the push and recovery phase of the forces on the shoulder joint.

The increments in biomechanical parameters for each protocol were analyzed and they were higher in protocol A for all the parameters except for lateral peak force, and for peak adduction and abduction moments (Table 4). Figures 5 and 6 show representative mean cycle of shoulder joint forces and moments data, respectively, for the group analyzed for both protocols.

**Table 4:** **Intra-protocol differences (early and late propulsion) for peak forces (N) and moments (N⋅m) acting on the shoulder joint, mean (SD)**.

	SCI subjects			
	High-intensity task	Low-intensity task	Inter-protocols	*p*-Value
Cadence	−0.01 (0.15)	−0.05 (0.12)	0.03	0.50
Fx (+anterior, −posterior)
Max	11.07 (12.98)	−2.04 (3.52)	13.11	**<0.01**
Min	−38.94 (18.60)	−2.70 (8.77)	−36.23	**<0.01**
Fy (+superior, −inferior)
Max	21.14 (19.41)	−0.59 (7.55)	21.74	**<0.01**
Min	−19.91 (27.46)	−4.09 (6.82)	−15.81	**<0.05**
Fz (+lateral, −medial)
Max	4.81 (8.23)	1.21 (5.66)	3.59	0.19
Min	−5.65 (7.90)	0.73 (3.77)	−6.38	**<0.05**
Mx (+adduction, −abduction)
Max	2.80 (5.94)	−0.03 (1.62)	3.11	0.07
Min	−2.63 (4.67)	−0.20 (0.77)	−2.43	0.06
My (+int.rotation, −ext.rotation)
Max	2.47 (2.08)	−0.02 (0.65)	2.49	**<0.01**
Min	−2.45 (2.94)	−0.02 (0.79)	−2.42	**<0.01**
Mz (+flexion, −extension)
Max	12.16 (7.37)	0.82 (2.43)	11.34	**<0.01**
Min	−4.89 (7.61)	−0.62 (1.15)	−4.26	**<0.05**

*Statistical significance (*p*-value) is related to the inter-protocol differences*.

*Bold font indicates statistical significance at *p* < 0.05*.

### Shoulder biomechanics and ultrasound parameters

No differences were found for the ultrasound parameters before and after each protocol (Table 5). Regarding the correlations between changes in kinetic and ultrasound findings before and after protocol A, increases in medial peak shoulder force were correlated with increases in LBTT (ρ = 0.594, *p* < 0.05) and with decreases in subacromial space measured following Cholewinsk index (ρ = −0.534, *p* < 0.05) (Table 6).

**Table 5:** **Mean (SD) ultrasound values before and after wheelchair propulsion tasks**.

	High-intensity task			Low-intensity task		
	Before	After	*p*-Value	Before	After	*p*-Value
LBTT	0.41 (0.09)	0.42 (0.07)	0.86	0.40 (0.09)	0.40 (0.06)	0.90
LBS	4.03 (0.66)	3.75 (0.93)	0.35	4.27 (0.65)	4.3 (0.87)	0.92
ACD	0.66 (0.16)	0.70 (0.15)	0.52	0.71 (0.15)	0.75 (0.15)	0.56
CHI	2.46 (0.45)	2.35 (0.59)	0.55	2.42 (0.49)	2.39 (0.51)	0.87
SST	0.64 (0.08)	0.61 (0.08)	0.48	0.62 (0.06)	0.60 (0.07)	0.56
SSS	4.41 (0.54)	4.42 (0.44)	0.96	4.46 (0.71)	4.30 (0.72)	0.55

*LBTT, long-axis biceps tendon thickness; LBS, long-axis biceps sonoelasticity; ACD, acromioclavicular distance; CHI, Cholewinski index; SST, short-axis supraspinatus thickness; SSS, short-axis supraspinatus sonoelasticity*.

**Table 6:** **Correlation between shoulder joint kinetics and ultrasound variables considering the changes in each protocol**.

	Fymin (inferior)		Fzmax (lateral)		Fzmin (medial)		Mxmax (adduction)	
	R. spear	*p*	R. spear	*p*	R. spear	*p*	R. spear	*p*
High-intensity task
LBTT	0.554	<0.05			0.594	<0.05		
CHI					−0.534	<0.05		
Low-intensity task
SST			0.538	<0.05	0.574	<0.05	0.578	<0.05

*LBTT, long-axis biceps tendon thickness; CHI, Cholewinski index; SST, short-axis supraspinatus thickness*.

## Discussion

To the best of our knowledge, this is the first study to address changes in shoulder joint kinetics with anatomical shoulder soft tissue changes recorded in ultrasound images after performing two different propulsion tasks that exert stronger or weaker physical demands. In accordance with our hypothesis, shoulder joint forces were stronger in the more intense manual propulsion task (protocol A) with respect to the less intense (protocol B). However, no differences were found for the ultrasound parameters before and after each propulsion task.

Regarding the kinetic variables, these are difficult to compare directly with data in the literature due to the different testing procedures, units of measurement, equipment employed, and characteristics of the population studied (Gil-Agudo et al., 2010a). A high-intensity wheelchair propulsion test was chosen considering that greater shoulder joint forces and moments were more likely to provoke shoulder pathology (Mercer et al., 2006). Moreover, such pathological changes might be easier to detect with ultrasound examination. Owing to the use of a treadmill for the experimental set up, we modified the resistance to be overcome by the subject by increasing the weight attached to a pulley system, a method that is safer than increasing the treadmill speed. However, we considered it interesting to compare these results with a lower intensity protocol that cause weaker shoulder joint forces and to correlate these with ultrasound findings.

Thus, we analyzed two specific workload settings and in both protocols, treadmill speed was individualized in order to normalize the power demand for all subjects to 20 W and this speed remained constant in both protocols. From our previous experience, we choose to develop a high-intensity wheelchair propulsion test on a treadmill without increasing the slope for safety and mechanical reasons (Hartung et al., 1993). Increasing the speed of the treadmill might cause heterogeneous increases on the loads that the subject has to overcome depending on its own weight. Therefore, to normalize the power to be overcome by the subject, we employed a procedure that calculates the increasing weights to be imposed by a pulley system in order to normalize every 5 W increase in this incremental test. For the low-intensity propulsion task, the speed and weight required to propel at 20 W remained constant during 20 min adapted from a previous long-term wheelchair propulsion protocol (Gass et al., 1981).

In the present study, the more intensive task produced increases in all directions of shoulder joint forces and almost all moments, as found previously when increasing speed (Mercer et al., 2006; Collinger et al., 2008). The greater posterior and lateral shoulder forces were previously related to pathological findings (Mercer et al., 2006). However, we did not find an increase in the LBTT after the high-intensity task, in contrast to a previous report (Van Drongelen et al., 2007), probably because our test was performed in less time. The subjects included in our study were experienced in manual wheelchair propulsion (time since injury 90.2 ± 54.78 months) and it is likely that the participants were accustomed to such exercise, possibly explaining why no differences could be detected in the ultrasound parameters before and after the propulsion tests.

With respect to the relation between kinetic and ultrasound findings, we found that in high-intensity protocols long-biceps tendon thickness increases when medial and inferior forces increases. Also, subacromial space measured following Cholewinsk index decreases when shoulder medial forces increases. Subjects need to propel the wheelchair everyday. So, instead of limiting subject’s activity, the need is to reduce the overall force to propel the wheelchair by accomplishing an alternative wheelchair set up or propelling with different technique. Bigger changes after high-intensity protocol were expected in relation to low-intensity task because the amount of work might be a risk factor for developing overuse injuries.

Although the biceps tendon diameter was similar to the reported elsewhere, those results are not directly comparable since we focused on potentially pathological parameters, such as decreased tendon thickness rather than other parameters like echogenicity (Van Drongelen et al., 2007). Similarly, we did not use grayscale-based quantitative ultrasound (Collinger et al., 2010), which may provide indicators of more microscopic damage. A lower echogenicity ratio of the biceps tendon has been reported, which might indicate the presence of a shoulder pathology after exercise but not an increase in biceps tendon diameter (Van Drongelen et al., 2007). Grayscale-based quantitative ultrasound proved to be useful to study the development of repetitive strain shoulder injury, although the appearance of supraspinatus post-propulsion was not significantly influenced by the biomechanics of propulsion (Collinger et al., 2010). We did not find differences in ultrasound images before and after both propulsion tasks in reference to the anatomic shoulder references and macroscopic tendon characteristics. We expected that more evident shoulder ultrasound changes would be produced by the high-intensity workload imposed. However, we also considered that the changes in the characteristics of the biceps and supraspinatus tendons are not only directly related to wheelchair propulsion but also the amount of change was related to the specific workload (Van Drongelen et al., 2007). Indeed, it appears that risk of developing shoulder joint damage is higher in subjects with long-term SCI using a wheelchair than after a high-intensity wheelchair propulsion task (Akbar et al., 2010). However, some correlation between shoulder kinetics and ultrasound images has been shown.

No relationship has been found between pain and imaging abnormalities (Boninger et al., 2001) and we agree that pathological findings in ultrasound images are not necessarily symptomatic, and thus, we also believe risk factors for clinical pathology should be identified before the individual becomes symptomatic (Mercer et al., 2006).

One limitation of this study was the small sample size and likewise, the number of subjects with and without shoulder pain. This limitation prevented us from performing a comparative analysis, and assessing the correlations between clinical data and kinetic or ultrasound findings. It should be noted that pain may confound the relationship between propulsion and ultrasound variables. In any case, we consider the findings presented here to be of interest considering that correlations between shoulder joint kinetics and ultrasound examination before and immediately after a propulsion task are a novelty itself. Nevertheless, further research will be necessary to identify relationships between kinetic data, ultrasound parameters, and clinical findings.

## Conclusion

Shoulder joint forces and moments increase in an intense propulsion task allowing the relationship between intensity and loads on the development of shoulder pain to be seen. However, no differences were found in ultrasound images after a high-intensity wheelchair propulsion task was carried out. More research is needed to collect clinical information and correlate data on shoulder pain with ultrasound images and kinetic information.

## Conflict of Interest Statement

The authors declare that the research was conducted in the absence of any commercial or financial relationships that could be construed as a potential conflict of interest.
